# The Usefulness of Cremation

**Published:** 1878-10

**Authors:** 


The Usefulness of Cremation.—Dr. LeMoyne, of Washington, Pa., takes advantage of the Southern epidemic to say a word in favor of his pet theory of cremation. It will be remembered that he has built the only crematory in this country. "Burial at any place" says the doctor, "is followed by baneful results, but a shallow grave in the sandy soil of the South, which is no absorbent, and cannot neutralize the gases, which commence to float up at once, is especially so, and more especially where the person dies of such a disease as the yellow fever. By cremation, while they dispose of the bodies more quickly than by any other mode, they at the same time purify"

He had just received a letter, he said, from Indianapolis, stating that a club had been formed in that city, having one hundred members from the first, and were now about to erect a crematory. Dr. Fletcher, one of the oldest physicians of the city, is at the head of it. The doctor gives it as his observation, as well as that of others interested in the matter, that a majority of the converts to the reform are of the gentler sex.—*Philadelphia Medical and Surgical Reporter.*

